# An Analysis of Predator Selection to Affect Aposematic Coloration in a Poison Frog Species

**DOI:** 10.1371/journal.pone.0130571

**Published:** 2015-06-25

**Authors:** Corinna E. Dreher, Molly E. Cummings, Heike Pröhl

**Affiliations:** 1 Institute of Zoology, University of Veterinary Medicine Hannover, Foundation, Hannover, Germany; 2 Department of Integrative Biology, University of Texas, Austin, TX, United States of America; University of Sussex, UNITED KINGDOM

## Abstract

Natural selection is widely noted to drive divergence of phenotypic traits. Predation pressure can facilitate morphological divergence, for example the evolution of both cryptic and conspicuous coloration in animals. In this context Dendrobatid frogs have been used to study evolutionary forces inducing diversity in protective coloration. The polytypic strawberry poison frog (*Oophaga pumilio*) shows strong divergence in aposematic coloration among populations. To investigate whether predation pressure is important for color divergence among populations of *O*. *pumilio* we selected four mainland populations and two island populations from Costa Rica and Panama. Spectrometric measurements of body coloration were used to calculate color and brightness contrasts of frogs as an indicator of conspicuousness for the visual systems of several potential predators (avian, crab and snake) and a conspecific observer. Additionally, we conducted experiments using clay model frogs of different coloration to investigate whether the local coloration of frogs is better protected than non-local color morphs, and if predator communities vary among populations. Overall predation risk differed strongly among populations and interestingly was higher on the two island populations. Imprints on clay models indicated that birds are the main predators while attacks of other predators were rare. Furthermore, clay models of local coloration were equally likely to be attacked as those of non-local coloration. Overall conspicuousness (and brightness contrast) of local frogs was positively correlated with attack rates by birds across populations. Together with results from earlier studies we conclude that conspicuousness honestly indicates toxicity to avian predators. The different coloration patterns among populations of strawberry poison frogs in combination with behavior and toxicity might integrate into equally efficient anti-predator strategies depending on local predation and other ecological factors.

## Introduction

A successful predation event consists of two elements: detection of prey and realization of an attack [1]. Aposematism and crypsis are two anti-predator strategies, which hinder successful predation on prey at different stages of the predation event: crypsis aims to prevent successful detection of prey by blending into the background [2], while aposematism signals unprofitability of prey to a predator via conspicuous traits associated with unpalatability [3, 4]. Depending on local conditions (e.g. habitat heterogeneity, predation pressure, and availability of noxious prey), different anti-predator strategies might be of selective advantage for a species. Furthermore, phenotypic signals, which are perceptible to heterospecific observers like predators might also be used in intraspecific communication (e.g. in mate selection and intrasexual aggressive interactions), and their evolution might be affected by several selective forces simultaneously [4–9].

Environmental conditions are likely to differ among geographically isolated populations, and in different populations opposing expressions of a signal may be of selective advantage. Interactions of natural selection with sexual selection and stochastic processes might furthermore contribute to divergent evolution of phenotypic traits among different populations of a species. For example, in several fish species (*Poecilia reticulata*, *Xiphophorus helleri*, *Alticus arnoldorum*), the evolution of elaborate signals face a trade-off, since they attract both mates and increase the risk of attacks by predators [7, 9, 10]. In the aposematic wood tiger moth, *Parasemia plantaginis*, the maintenance of polymorphism is the result of a trade-off between predator selection and mating success: white males had higher mating success while yellow males survived better when confronted with predators [11]. On the other hand, natural and sexual selection might act in concert on the evolution of phenotypic features, e.g. in *Heliconius* butterflies coloration and spotting pattern seem to be optimized for both predator deterrence and mate attraction [6]. Some species of aposematic dendrobatid frogs show high variability of coloration among populations. In *Ranitomeya imitator*, frogs of different populations use Müllerian mimicry and resemble several other sympatrically occurring toxic frog species in coloration and pattern (e.g. *R*. *ventrimaculata* and *R*. *fantastica*) [12]. In the polytypic species *Oophaga granulifera*, rather cryptic and rather conspicuously colored populations have been described [13], whose divergence might have been facilitated by natural selection via differences in predation pressure among populations [14].

In strawberry poison frogs (*Oophaga pumilio*), skin coloration and patterning is highly diverse among populations, comprising more than 15 distinct, mostly geographically separated, color morphs [15–17]. Most color morphs are located at the islands and adjacent mainland of the Bocas del Toro Archipelago in Panama, despite the very recent formation of the Archipelago in the last 10,000 years [18]. Strawberry poison frogs are a popular model organism for research addressing questions about intra- and interspecific communication. They produce signals of several modalities, including visual and acoustic signals which vary geographically [16, 19]. Color divergence among populations of *O*. *pumilio* has been proposed to be driven by both sexual and natural selection (see reviews [5, 20]).

As the aposematic function of conspicuously colored populations of *O*. *pumilio* has been confirmed by several experiments, predation was suggested to have played a substantial role in this divergence [21–23]. Although the conspicuousness of several polytypic populations reliably indicates their toxicity to predators [22], several populations of cryptic dull coloration exist [24]. In two populations of *O*. *pumilio*, the cryptic and aposematic anti-predator strategy was further supplemented by the respective inconspicuous or bold behavior of the frogs [25]. If natural selection exerted by predators has been important for the formation of color morphs in *O*. *pumilio*, we expect predation pressures to vary among populations [21, 26]. Several previous projects used clay model frogs to evaluate predation pressure on *O*. *pumilio* [1, 21, 23, 27] and other dendrobatid frog species [14, 28, 29]. In *Ranitomeya imitator* and *Dendrobates tinctorius*, predation caused by birds was significantly higher on novel aposematic morphs compared to local aposematic morphs, which exerts strong homogenizing selection in favor of local color morphs [28, 30], in particular in monomorphic populations [29]. Red coloration is considered to be an effective aposematic signal [31], and red clay models were less attacked than brown controls in a red population of *O*. *pumilio* [23]. To the opposite, in a cryptic population [25], clay models of local coloration were more attacked than non-local aposematic ones and brown controls [21]. Despite these previous studies it is still unknown how predation pressure and conspicuousness of the frogs to a variety of predators with different visual systems interact on a larger geographic scale covering multiple frog color morphs. Here we use two approaches to understand the interplay between predation pressure and the conspicuousness of the frogs. First, we applied visual modeling to explore if frogs of local coloration are particularly conspicuous or particularly cryptic to certain predators in comparison to their conspicuousness to conspecifics which indicates their importance for sexual selection. Second, using clay model frogs of four different colors (including the color of the local frog population) we investigated 1) which animals may be major predators of strawberry poison frogs 2) if predation pressure and the composition of predator communities vary among populations, 3) whether predation differs among clay frogs of different colors, especially among local and non-local colors. We furthermore interpret our predation data to tackle the question whether conspicuousness of the live frogs and attacks by (certain) predators co-vary. Thus our study combines information about predation pressure caused by local predators and the visual conspicuousness of living specimens of local strawberry poison frogs to different predator classes.

## Material and Methods

### Reflectance Measurements and Visual Modeling

Field work was conducted between December 2008 and June 2011 in six populations of strawberry poison frogs, two populations in Costa Rica and four populations in Panama. To assess the conspicuousness of the frogs on their specific substrate for conspecific and several heterospecific observers we took reflectance measurements. We measured the spectral reflectance of the skin of a total 255 frogs in Sarapiquí (n = 40 red frogs; 10° 28.227 'N; 84° 0.553 'W; 44 m.a.s.l.), Hitoy Cerere (n = 52 red frogs; 9° 37.819 'N; 83° 0.879 'W; 270 m.a.s.l.) (both Costa Rica), Río Gloria (n = 38 yellow frogs,; 8° 59.100 'N; 82° 13.916 'W; 24 m.a.s.l.), Tierra Oscura (n = 47 blue frogs; 9° 11.776 'N; 82° 14.976 'W; 7 m.a.s.l.), Isla Colón (n = 40 green frogs; 9° 23.170 'N; 82° 15.941 'W; 35 m.a.s.l.) and Isla Solarte (n = 38 orange-red frogs; 9° 19.946 'N; 82° 12.939 'W; 4 m.a.s.l.) (Panama). Additionally we measured the reflectance of the specific substrate (e.g. leaves, trunks) on which each individual frog was found. Reflectance spectra of the skin and substrate were taken at a distance of 2mm using an Ocean Optics HR2000+ Spectrometer, an Ocean Optics bifurcal optic fiber (R-200-7-UV/VIS) with a fixed outer sleeve to control for the 2mm distance and a deuterium-tungsten lamp (DT-Mini-2-GS). To account for lamp drift we calibrated the measurements with a white standard (WS-1-SS) every other frog. Illumination of the habitat (Irradiance) was measured using an optic fibre (QP400-2-UV-BX) with an Ocean Optics cosine adaptor-head (CC-3UV). Irradiance spectra were taken at the places where we found the frogs and at times when the frogs showed most activity (between 7am and 12noon in Costa Rica; and between 8am and 1pm in Panama) on two to three different days. The population-specific average irradiance was calculated for each population, including between 192 and 396 irradiance spectra per population.

We calculated average reflectance spectra for dorsal and ventral regions for each frog. For dorsal reflectance spectra four reflectance measurements were averaged (two of which were taken on the head between the eyes, and two on the middle of the dorsum). For frogs from the population of Río Gloria and on Isla Colón, where frogs possess a dark spotting pattern on a yellow or green background color on their dorsums, we included two measurements of the dorsal background color and the two head measurements for dorsal average spectra. We neglected the influence of dark spots, because spotting pattern did not affect predation risk on clay models of *O*. *pumilio* in an earlier study [32], and bird predators were found to base attack decisions on coloration cues rather than on contrasting patterns [33–35]. For calculations of ventral measurements we averaged two reflectance curves taken on the belly. We did not include measurements taken from the throat region in order to avoid the darker coloration of the throat of males to impact the results. Because there is no general sexual dimorphism described in this species [36], and our study does not focus on differences between males and females, we analyzed males and females together. An equal number of males and females was measured in each population.

Visual models were calculated according to Maan & Cummings and Crothers & Cummings [22, 37] using average dorsal and ventral reflectance spectra from each frog, from the specific substrate of each frog and the population-specific average irradiance. For the trichromatic visual model of a conspecific viewer, microspectrophotometric data on the visual sensitivity of cones of *O*. *pumilio* [38] were used. For avian, crab, and snake visual models we used data of visual sensitivity of the European Starling (*Sturnus vulgaris*, [39]), the fiddler crab (*Uca tangeri*, [40–42]) and the Coachwhip (*Masticophis flagellum*, [43, 44]). These predators differ in their visual systems, including dichromatic, trichromatic and tetrachromatic vision. The crab vision model was calculated for the dichromatic visual system of *Uca tangeri* [41, 42]. The trichromatic visual models for snakes and *O*. *pumilio* include information about the spectral sensitivity of *Masticophis flagellum* [43] and one specimen of *O*. *pumilio* [38]. The avian visual model we used is based on a tetrachromatic system with UVS-cones and brightness contrast of this model was calculated using the spectral sensitivity of double cones with oil droplets (as in [39]). For these four observers, we calculated brightness contrast (ΔL) and color contrast (ΔS) for each frog on its specific substrate. Brightness contrast yields negative results, if frogs are darker than their specific background. Since we are interested in their conspicuousness in terms of how they contrast to their substrate rather than if they are brighter or darker than the substrate, we use the absolute values of brightness contrasts. Finally we estimated the dorsal and ventral overall conspicuousness of the frogs on their substrates for the visual models of birds, snakes and crabs. Overall conspicuousness between frog and background was calculated as the Euclidean distance ($OC=\sqrt{△S^{2}+△L^{2}}$) based on the average color and brightness contrast for every population as proposed by Cummings & Crothers [5].

Our study species is not a species protected by the laws of Panama or Costa Rica. However it is on the CITES Appendix II colourful dendrobatid species are often collected illegally for commercial reasons. *Oophaga pumilio* is abundant across its range and listed as a species of "least concern" by the IUCN. All sampling and measuring methods are described in detail above. No animals have been sacrificed and all individuals have been released at their capture site after measuring. Approval by an ethics committee was not necessary since all sampling methods and manipulation of the frogs is part of the evaluation of the study by the local authorities (SINAC and ANAM) that authorize the research permits.

### Data Analysis of Visual Modelling

Brightness and color contrast (ΔL and ΔS) of the frogs from the study populations were calculated for potential predators and conspecifics. As absolute values of color and brightness contrasts are not comparable among different visual models, i.e. different observers, we normalized the results of each calculated model and calculated means and standard deviation of color and brightness contrasts (ΔS and ΔL) for dorsal and ventral measurements for each population. We applied ANOVAs to test if contrasts differ significantly among populations for different (conspecific or heterospecific) observers. ANOVAs indicated differences in color and brightness contrasts of both body regions among populations for all observers. Therefore, we applied Tukey post-hoc tests to localize the differences among populations. We furthermore visually checked the residuals to justify the use of ANOVAs. Statistical analyses were conducted using STATISTICA 6.0.

### Predation experiments

To evaluate whether frogs of local or nonlocal (i.e. novel) colors are more or less prone to an attack, we conducted predation experiments with clay model frogs. The use of clay models as specimen replicates facilitates assigning damaged models to different predator categories, according to teeth marks or other characteristic imprints preserved in the clay [1]. Previous to the experiments, we measured the spectral reflectance of the clay colors used for clay models and compared the reflectance spectra of the clay models to those of real strawberry poison frogs of red, yellow, green and blue coloration, respectively. We considered the achieved accordance as sufficient for the experiments and used standard clay colors. Furthermore, we modeled one exemplary clay model frog, which was used to manufacture silicon molds. With these molds we were able to make model frogs of standardized shape in high numbers from non-toxic standard clay in four colors (red, yellow, green and blue). The experiments were conducted over time periods of 20 consecutive days, which were divided into four time intervals of five days. Per time interval, we placed 400 clay model frogs in the habitat of the frogs, resulting in a total number of 1600 clay frogs in each population. 400 clay frogs were put out simultaneously, in assorted groups of four frogs containing one frog of each coloration. In each population we selected two representative areas of the frog habitat, and placed 50 clay frog groups in consecutive transects of approximately 50 m in both areas. At the end of each time interval–after five days—the clay model frogs were controlled, and all damaged or missing frogs were replaced with new clay frogs. All predation marks were documented with pictures. Each clay model frog was scored as attacked or not-attacked and the attacked models were furthermore assigned to one of the following categories: bird marks (U- and V-shaped imprints), holes and scratches, rodent marks, snake marks, crab marks, lizard marks, missing models and unknown predation marks. Attacks assigned to the category holes and scratches were scored as potentially caused by birds, as they looked like the damages on clay frogs of the category bird marks, but missed the typical U- and V-shaped imprints [32]. Examples of attack marks assorted to the categories birds, holes & scratches, rodents, snakes, crabs, lizards and unknown predation marks are presented in S1 Fig We furthermore applied visual modeling to calculate color and brightness contrasts of the different colors of clay model frogs on typical substrates of each population. In these visual models, we included reflection of the clay model frogs, the population specific irradiance and the substrates of the living frog specimens of the respective population. All field experiments were conducted in accordance with the laws and ethical standards of Costa Rica and Panama. Research permits were obtained before starting field work from the local authorities.

### Data Analysis of Predation Experiments

There was no difference in frequency of predation among the consecutive time intervals of the experiment. Hence, for the analyses we summarized the attacks of all intervals. To analyze whether risk of an attack was predicted by the population, the coloration of the clay model frog and/or by its origin (local or non-local), we used Generalized Linear Models (GLM´s), assuming binomial-error distribution and a logit-link-function. Conducting deviance analyses with Chi-square statistics for the GLM´s we tested whether population, coloration and/or origin of the clay model were significant predictors for general risk of attack, and whether predation risk within each population was predicted by the latter two variables. Furthermore, we applied Tukey post-hoc tests to localize between which populations or clay model colors predation pressure differed. All analyses were calculated using R [45].

Considering the assignation of different predation marks to predator categories, our data–as well as previous studies [1, 22, 23]—strongly suggest, that birds are the main predators of *O*. *pumilio*. We therefore repeated the analyses considering only damages caused undoubtedly by birds (category ‘bird marks’: U- and V- shaped marks) and damages probably caused by birds (summing up the results of the categories ‘bird marks’ and ‘holes & scratches’ to a new category of ‘potential bird marks’). The assignment of ‘holes & scratches’ to ‘potential bird marks’ follows Hegna et al. [32] and is supported by the observations of Willink et al. [14], who observed birds causing similar attack marks. Because the majority of predation marks are assigned to this category of ‘potential bird marks’ and the low numbers of attacks for all other predator classes did not allow for statistical tests in these categories, we summarized all other predation marks to a new category ‘non-bird predation’ and ran the analyses for this category as well.

We furthermore tested, if predation pressure is correlated with high levels of conspicuousness (color and brightness contrasts and overall conspicuousness) of the local frogs, which would indicate that natural selection contributed to the evolution of aposematic coloration in this species. Correlation analyses were conducted with STATISTICA 6.0.

## Results

### Visual Modeling

Visual modelling calculations are based on dorsal and ventral reflectance spectra of differently colored frogs. Average reflectance spectra for each studied population are depicted in S2 Fig (Note the high spectral reflectance of the white ventral regions of some populations (e.g. Sarapiqui and Río Gloria) exceeded that of the lambertian white standard (Spectralon)).

### Color Contrast (ΔS)

**Fig 1:**
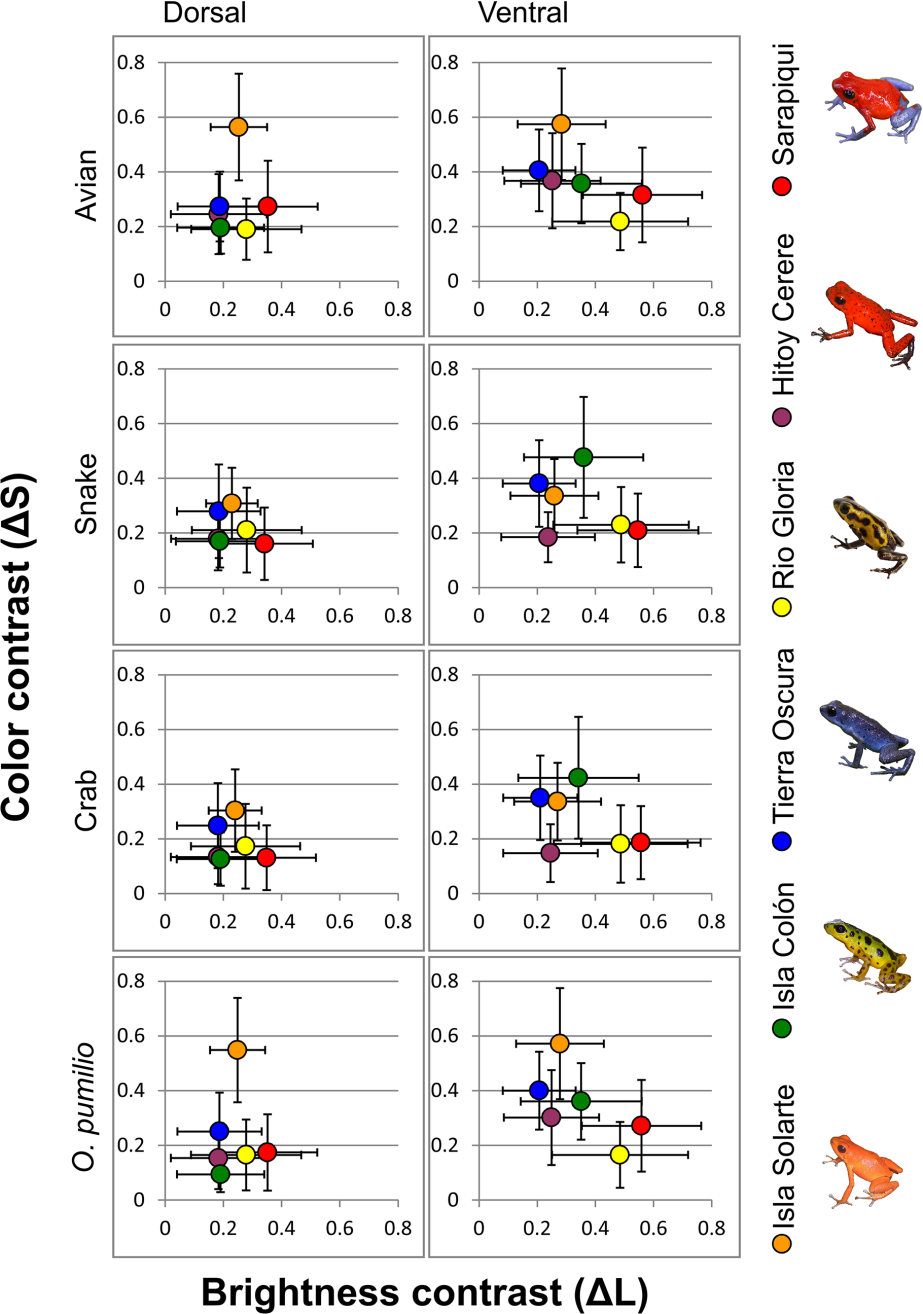
Visual conspicuousness of *O*. *pumilio* from the study populations for three different heterospecific (avian, snake and crab) and a conspecific observer. Graphs show average color and brightness contrasts of dorsal and ventral measurements of reflectance, normalized for each observer. Circles indicate mean values for brightness and color contrasts, bars show standard deviation of means for each population.

Dorsal contrasts: Visual modeling revealed that frogs from Isla Solarte had the highest values of dorsal color contrast (Fig 1). These contrasts were significantly higher than contrasts from all other populations for all observers, except for contrasts of frogs of Tierra Oscura for snake and crab vision (S1 Table). The dorsal color contrast of frogs from Sarapiquí, Hitoy Cerere, Río Gloria and Isla Colón were similar and lower than those from Isla Solarte and Tierra Oscura (Fig 1, S1 Table).

Ventral contrasts: For avian and conspecific vision, the frogs of Isla Solarte showed the highest values for ventral color contrast, which significantly differed from contrasts of frogs of most other populations, followed by contrasts of frogs from Tierra Oscura and Isla Colón. For avian and conspecific vision, frogs from Río Gloria had the lowest contrasts which were significantly different from most other populations. For crab and snake vision frogs from Isla Colón had the highest color contrasts, followed by frogs from Tierra Oscura and Isla Solarte, while frogs from Hitoy Cerere had the lowest contrasts (Fig 1, S1 Table).

### Brightness Contrast (ΔL)

Dorsal contrasts: Frogs of Sarapiquí displayed the highest dorsal brightness contrasts for all observers, and were significantly brighter than frogs from Hitoy Cerere, Tierra Oscura and Isla Colón, marginally brighter than frogs from Isla Solarte, but not significantly brighter than frogs from Río Gloria (Fig 1).

Ventral contrasts: Similar to their dorsal measurements, frogs from Sarapiquí had the highest ventral brightness contrasts for all observers, followed by frogs from Río Gloria. For all observers, brightness contrasts of frogs from Sarapiquí and Río Gloria were significantly higher than contrasts from all other populations. The frogs from Hitoy Cerere, Tierra Oscura and Isla Solarte displayed the lowest ventral brightness contrasts (Fig 1, S2 Table).

### Overall conspicuousness

Overall conspicuousness represents the combined contrast of Δ*L* and Δ*S* in a brightness and color contrast space (the Euclidean distance, see Fig 1). Dorsally the red (-orange) and yellow populations were more conspicuous than the blue and green ones for all observers. One exception was Hitoy Cerere where the frogs were red but duller than the red frogs from Sarapiquí and Isla Solarte. Ventrally the trend of conspicuousness was the same but the bright yellow ventral regions of the frogs from Isla Colón was equally or more conspicuous than the ventral regions of frogs from Isla Solarte.

### Contrasts of clay frogs

As for the live frogs the red and blue clay models showed a particularly high color contrast while the yellow models showed the highest brightness contrast for most predator—population combinations (S3 Fig).

### Predation experiments

**Fig 2:**
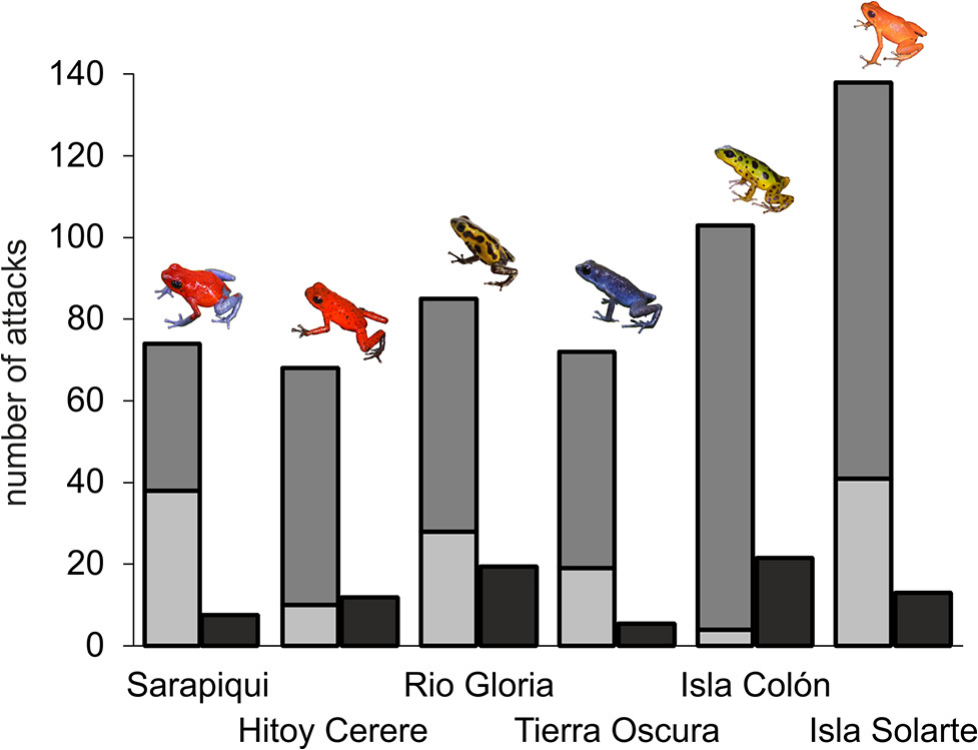
Number of predation events in each population. Bar coloration indicates the predator category the attack was assigned to. Light grey: U- and V-shaped attack marks caused by birds; grey: holes and scratches potentially caused by birds; and black: non-bird predation. Isla Colón and Isla Solarte are island populations, while all others are located on the main land.

**Fig 3:**
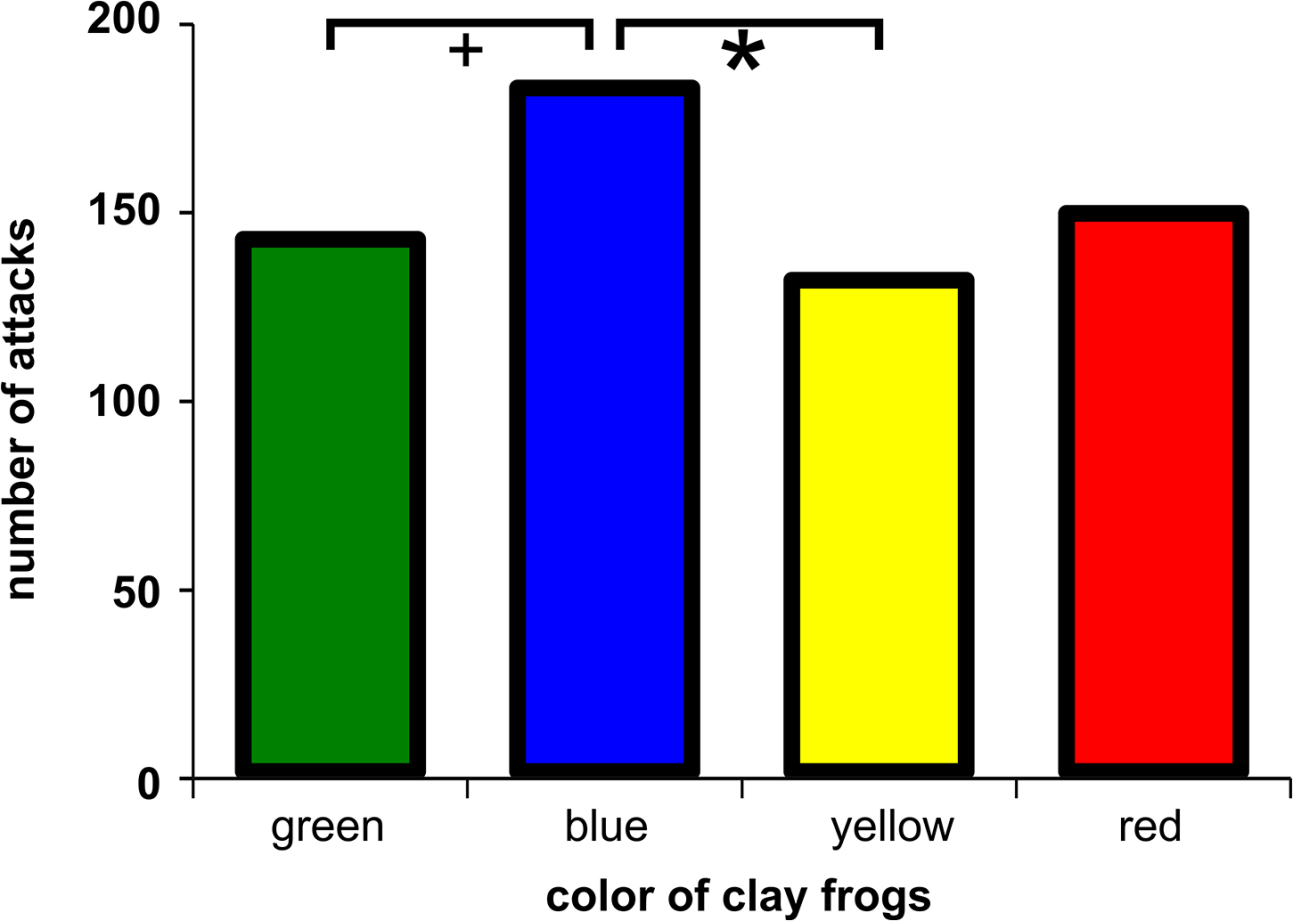
Predation on clay frogs of different colors. Overall number of attacks on clay frogs from all six studied populations of *O*. *pumilio*. **+** P < 0.10; * P < 0.05.

We found a highly significant difference in overall attack rate among populations and a marginally significant effect of clay model color, while we did not detect any significant effect of the origin (local or non-local) of the model frogs (Table 1, S3 Table). Overall attack rate on clay models in both island populations in Panama (Isla Colón and Isla Solarte) was significantly higher than overall attack rate in Sarapiquí, Hitoy and Tierra Oscura mainly due to attack by ‘potential bird marks’ (Fig 2). Attack rate on blue clay frogs was significantly higher than on yellow frogs (Fig 3). In Sarapiquí, attack rate on blue frogs was higher than on yellow and on green frogs (Table b in S4 Table).

**Table 1:** Effects of frog population, clay frogs coloration and origin on the attack rate of different predator on clay frogs.

	Overall attack rate	bird marks	potential bird marks	non-bird predation
Population	**0.0000**	**0.0000**	**0.0000**	**0.0108**
Clay model color	**0.0162**	**0.0029**	**0.0298**	0.1760
Origin	0.9214	0.8195	0.5476	0.0857

Results (P-values) of generalized linear models (GLM´s), which tested whether there was an effect of population, coloration of clay frogs or clay frog origin (local versus non-local coloration) on the attack rate of different predators.

The results strongly suggest that birds are the main predators of *O*. *pumilio* (Fig 2, S3 Table). In the category ‘bird marks’ population and clay model color were significant predictors of attack rate (Table 1). In this category more attacks on clay frogs were observed in the yellow to red populations Río Gloria, Isla Solarte and Sarapiquí (but not on clay frogs in Hitoy Cerere). Attack rate in Isla Colón was significantly lower than in Sarapiquí, Río Gloria, Tierra Oscura and Isla Solarte. Furthermore attack rate in Hitoy was significantly lower than in Sarapiquí, Río Gloria and Isla Solarte (Table c in S4 Table). We observed more ‘bird marks’ on blue than on yellow or green clay frogs in *O*. *pumilio*, i.e. across all populations, and in Sarapiquí (Fig 3, Table d in S4 Table).

The analysis of ‘potential bird marks’ yielded similar results. Overall there was a highly significant effect of population and a marginally significant effect of clay model color on predation risk (Table 1). Predation risk on Isla Solarte was significantly different from all other populations except from Isla Colón (Table e in S4 Table). Overall predation rate on blue clay frogs was significantly higher than on yellow and green frogs (Table f in S4 Table).

There were low numbers of attacks, which were assigned to other predators like crabs, snakes and lizards or as ‘missing’ and ‘unknown’ (S3 Table). All these attacks were analyzed together in the category ‘non-bird predation’ (Fig 2). There was no detectable effect of clay model color or origin on attack rate (Table 1, Table h in S4 Table). While there was an overall effect of population on attack rate (Table 1), pairwise comparisons of populations only detected a trend for a difference in attack rate between Isla Colón and Tierra Oscura (P = 0.08) (Table g in S4 Table). Even though the number of non-bird predation events is very low, Isla Colón is the only population with 4 documented snake and 2 documented crab attacks (Hitoy Cerere: two lizards and one crab; Isla Solarte: two snakes). In Tierra Oscura no recorded attack was assigned to non-bird predation.

### Correlation between conspicuousness of local frogs and risk of attack

**Fig 4:**
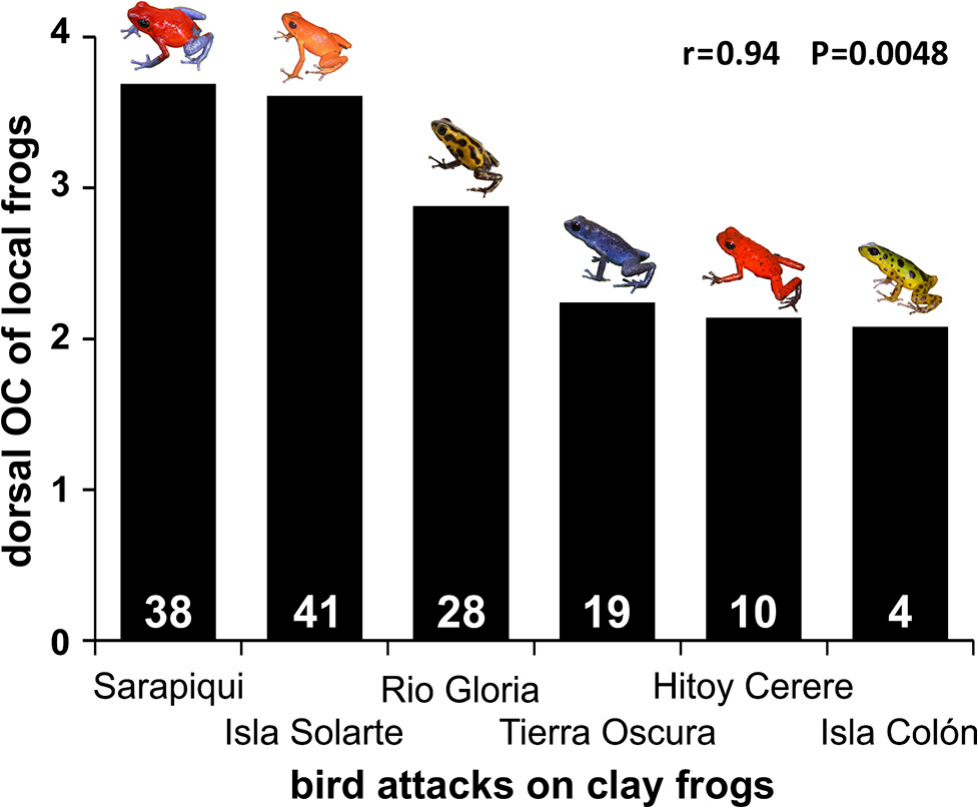
Correlation between dorsal overall conspicuousness of local frogs for avian eyes and avian predation on clay frogs across frog populations. The correlation is highly significant (r = Spearman rank correlation coefficient).

Across populations, the dorsal brightness contrast of the local frogs for bird eyes was highly, almost significantly correlated with the total number of attacks by birds on clay frogs (r = 0.79, P = 0.06) (S5 Table). Total number of attacks by birds includes attacks on all clay model colors of variable conspicuousness to bird predators, and thus estimates avian predation pressure in each population. Importantly the only highly significant correlation between attack rates on clay frogs and conspicuousness of the local frogs was between the dorsal overall conspicuousness for the bird visual model and number of bird attacks (r = 0.94, P = 0.0048; N = 6, Fig 4). Dorsal color contrast, ventral color and brightness contrasts as well as ventral overall conspicuousness of frogs did not show any association with attack rates (data not shown).

## Discussion

Our study yielded several interesting results. Strawberry poison frogs from populations differed in their coloration and conspicuousness, i.e. in color and brightness contrast as well as in overall conspicuousness. In predation experiments with clay model frogs, predation risk varied among frog populations and was higher on island than on mainland populations. Birds were the main predators, while attacks of other predators like lizards, snakes, rodents and crabs were rare. Unlike other studies, our results do not provide evidence that the local color morph was better protected than other color morphs. However bird predation was highest on blue frog models (a non-local color morph in five of the six populations of our study). Our finding of higher attacks on blue frog models than on yellow or green models might indicate that frogs of high conspicuousness (yellow) or low conspicuousness (green) were better protected than intermediate color morphs (blue). Moreover, in populations where avian predation on clay frogs was higher, local frogs were dorsally brighter and more conspicuous, which suggests that predation exerted by birds primarily selects for higher conspicuousness in the local prey.

### Conspicuousness and toxicity

The results of visual modeling demonstrate that contrasts calculated for each population are very similar among different observers especially between snake and crab and between avian and *O*. *pumilio* visual models for the specific background where each frog was found. For all observers, including the conspecific viewer, frogs from the population of Isla Solarte possess the highest dorsal color contrasts on their substrates from all studied populations but also possess a high brightness contrast and overall conspicuousness. Previous studies revealed that Isla Solarte is one of the most toxic populations of *O*. *pumilio* [22]. Being highly toxic and conspicuous [22], Isla Solarte frogs employ an aposematic anti-predator strategy, which is also reflected in their bold behavior [25, 36]. The study of Maan and Cummings (2012) revealed a positive correlation between brightness and overall conspicuousness to bird eyes and the noxiousness of frogs in 10 different Panamanian populations [22], i.e. toxicity seems to be honestly signaled by the dorsal coloration of the frogs. A theoretical framework presented by Holen and Svennungsen [46] suggests that honest signaling in the tradition of the handicap theory can potentially be achieved by two mechanisms: the “go slow” behavior of predators or the “resource allocation trade-off” in prey. In “go slow” predators are reluctant when tasting conspicuous and well defended prey. Survival of conspicuous prey is therefore enhanced. The “resource allocation trade-off” hypothesis instead proposes that the same resource is involved in the expression of the warning signal and the defense (e.g. toxicity). In case the resource is limited only individuals with access to a high resource quantity can afford to develop conspicuous signals. Conspicuous warning signals improve predator learning and as a consequence the number of attacks decreases. Currently it is unknown whether one of these mechanisms applies to the evolution of aposematism in our study model.

An additional possibility is the involvement of sexual selection. Our data reveal that avian predators and conspecifics, i.e. potential mating partners, perceive frogs as similarly conspicuous when evaluating contrasts against the specific backgrounds and irradiance conditions in which the frogs were captured. While sexual selection often favors high conspicuousness, it entails easy detection by predators. This might imply that sexual selection for brighter colors in strawberry poison frogs evolved in concert with higher toxicity reinforcing aposematism in this species. The interaction between natural and sexual selection for the divergence in warning colors in this and other taxa warrants further study [5].

### Predation risk

Our experiments with clay model frogs showed, that predation pressure significantly varies among populations with higher predation rates on islands (Isla Solarte and Isla Colón) compared to mainland populations. These results are contrary to the general assumption of relaxed predation pressure on islands [47, 48]. For spiny-tailed iguanas [49] and nest predation of several bird species experiments confirmed less predation on island populations compared to mainland populations [49–51], however, one study conducted in Sweden also found predation pressure on nests of two bird species to be equal for island and mainland study sites [52]. In this regard our study does not support the hypothesis, that relaxed predation pressure in the Bocas del Toro region allowed the evolution of a high diversity of warning signals in strawberry poison frogs, as proposed by Hegna et al. [21]. However, our results strongly suggest that birds are the main predators of *O*. *pumilio*. Damages caused by crabs or snakes or other unidentified animals were rare. Most predation events on Isla Solarte clearly resulted from birds, but on Isla Colón more attacks were assigned to the category “holes and scratches”. In this regard more research is necessary to confirm the true nature of these predators and to differentiate predator composition among populations. One previous study using clay model frogs to investigate predation pressure in *O*. *pumilio* on Isla Colón confirmed that even though overall attack rate was high on the local frogs, unambiguous bird predation was low on this island compared to mainland populations [21]. We obtained lower overall predation rates (4.8–7.7%) in comparison to similar experimental studies with clay frogs in this species (7.5–12.6%) [21, 31, 32], which might be due to differences in localities, duration of the experiments as well as shape and color of clay frogs.

### Predation on local versus non-local coloration

Our data does not provide direct evidence that model frogs of local coloration are better protected than model frogs of non-local coloration. The color which was most attacked in all four mainland populations (Sarapiquí, Hitoy, Río Gloria and Tierra Oscura) was blue, which is a non-local color for all populations except Tierra Oscura. In Tierra Oscura, where local frogs are blue and possess moderate toxicity levels (Aguacate population [5, 22]), local predators also attacked the blue morphs significantly more than green and yellow. Only in the population of Río Gloria the number of (bird) attacks was lowest on the yellow clay frogs of local origin. The low attack rate on yellow clay models might be due to a generally low number of attacks on yellow coloration–which was also found in Sarapiquí, Hitoy Cerere and Isla Solarte, rather than the local origin of the yellow coloration in this population. Since the yellow clay frogs are brighter than clay frogs of other coloration it is possible that predators—in this case mainly birds—avoid attacking the bright yellow objects since brightness reliably indicates toxicity in our study species. Similar studies in *O*. *pumilio* found that attack rates on red and yellow clay frogs were not affected by the local frog coloration [31] or found clay frogs of local coloration to be more attacked than novel brown or red models in one of our study populations (Isla Colón) [21]. On the contrary, studies of predation pressure on clay frog models of local or non-local origin in two other dendrobatid frog species (*Dendrobates tinctorius* and *Ranitomeya imitator*) reported that predation rate on local color morphs was lower than on a (conspicuous) novel color morph [29, 30]. An explanation of these different findings might be that in the latter studies predation experiments were finished after 72 hours. Nonetheless the study on *R*. *imitator* showed that the initial advantage of the local color morphs disappeared during the experiment and after 72 hours all frog morphs in the experiment were equally attacked. Thus the apparent local color morph advantage is of short duration, which makes a long-term evolutionary effect unlikely.

Overall, our data do not provide evidence for substantial differences of predation pressure on clay model frogs as a function of the local coloration of strawberry poison frogs. Three possible explanations for this result are as follows: First, predation pressure might not be the major force driving divergence in coloration among populations and its contribution to divergence in coloration might be concealed by the impact of other forces (i.e. toxicity levels and sexual selection). Second, selection for divergence in color patterns cannot be examined properly as predator communities may have changed since color morphs diverged among different populations in the Bocas del Toro archipelago [31] and elsewhere, as proposed by Alcover and McMinn [53]. Third, optimal local protection is not achieved by coloration alone but via a combination of coloration, behavior, body size and toxicity. This idea is supported by the finding that besides being less toxic, cryptic morphs are also smaller and use other anti-predator and reproductive strategies than more conspicuous morphs in strawberry poison frogs [25, 36, 54]. The differential availability of toxic food items (small insects that contain toxic alkaloids [55] among populations might also influence the evolution of conspicuous or cryptic coloration in *O*. *pumilio*.

### Correlation between conspicuousness of local frogs and attack rate

High conspicuousness (e.g. through high brightness contrast) can facilitate detection of prey by predators, but it might also provide protection through predator learning. Interestingly the rate of attacks which were unambiguously caused by birds is highly correlated with brightness contrast and overall conspicuousness of the local frogs for avian eyes. This indicates that bird predation selects for a bright, conspicuous coloration in local prey because the conspicuousness itself facilitates the education of birds for predator avoidance. For instance, unpalatable prey of higher brightness contrast provided greater predator aversion learning in the Chinese mantid than prey of lower brightness contrast [56]. However, we did not find the same correlation for ‘potential bird marks’. In this regard we need more clarity about the animal species, whose attacks have been categorized to this group. An erroneous classification of some damaged clay models as “potentially caused by birds” (e.g. in the category “holes & scratches”) might explain the missing correlation of “potential bird marks” and the conspicuousness of local frogs. Despite this finding conspicuous colors like orange, red and yellow, may generally function as effective aposematic signals for predator deterrence, even when predators are not familiar with frogs of these colors [31, 57].

### Comparison of predation risk among *Oophaga granulifera*, *O*. *pumilio* and other dendrobatid frog species

*Oophaga granulifera* is another dendrobatid frog species which shows color polytypism ranging from green to red populations in natural lowland forests along the pacific coast of Costa Rica and Panama [13]. This species is very closely related to *O*. *pumilio* and exhibits high similarity in its ecology, behavior and morphology. Research on *O*. *granulifera* explores the relation between predation pressure, toxicity and conspicuousness to the visual systems of birds with similar methods as in our study [13, 14, 58].

Contrary to *O*. *pumilio* [22], toxicity levels and visual conspicuousness were inversely related among populations of *O*. *granulifera*, i.e. green frogs were more cryptic but more toxic than red frogs [13], while the predator community was more diverse consisting of birds, lizards and crabs of similar magnitude [14]. Willink’s study revealed that predation in *O*. *granulifera* follows a specific pattern: birds avoided attacking clay models reproducing the local coloration of the frogs (i.e. red in populations of red frogs and green in populations with green frogs), while lizards mostly attacked red clay models which mimic the highly conspicuous but less toxic red frogs.

Another study, investigating the link among predator avoidance, visual conspicuousness and toxicity in three species of the genus *Epipedobates*, proposed that there might be a trade-off between conspicuousness and toxicity in some dendrobatid frogs [59]. A similar mechanism might apply to *O*. *granulifera* and different predator taxa might cause opponent selective advantages for phenotypic traits. For *O*. *pumilio* there is evidence that conspicuousness is an honest indicator of toxicity [22] for its main predator group. A more general avoidance of bright coloration might override the avoidance of the local frog coloration, since local frogs are not always highly toxic. The establishment of an honest signaling system might be facilitated when it is directed towards the sensory system of a single predator group. However, if various predator groups are involved alternative strategies might be favored which may even include a trade-off between conspicuousness and toxicity. Different predation patterns among *O*. *granulifera* and *O*. *pumilio* might furthermore be caused by differences in the ecology of these two species. *Oophaga granulifera* inhabits more natural, undisturbed habitats [60], where predator communities may not have changed in the last millennia. *Oophaga pumilio*, however, populates primary and secondary habitats, including abandoned agricultural areas (e.g. cocoa plantations).

### Future work

For a real understanding of the impact natural selection may have on color divergence of *O*. *pumilio*, further research is necessary. A persisting problem is the lack of knowledge about the actual predators of this species, which is essential for the evaluation of the importance of natural selection for color divergence [61]. Further studies including video traps [14], will help to evaluate, which animal taxa actually predate on strawberry poison frogs, and facilitate interpretation of damages inflicted on clay models. Afterwards, the evaluation of the importance of additional features (e.g. internal black spotting patterns, olfactory cues and movement) for prey detection and avoidance learning can be evaluated for different predator classes. To address the question of whether abundance of toxic prey might facilitate or constrain ongoing divergence of coloration in strawberry poison frogs, we recommend examining whether the availability of toxic prey differs among populations of strawberry poison frogs. We furthermore suggest comparing toxicity levels among different species of poison frogs. A comparison of the toxicity of *Oophaga granulifera* and *Oophaga pumilio* would allow setting the results of predation experiments and visual modeling in relation to toxicity levels. This may allow insights in trade-offs and the interplay between conspicuousness, toxicity and behavioral strategies and how these interactions may have affected color divergence of poison frogs. Finally, similar studies on other aposematic animals would help to achieve a better understanding of the relationship between “honest” or “dishonest signaling” and predator and prey ecology.

## Supporting Information

S1 Fig Examples of predation marks assigned to different categories: (a-c) birds marks; (d-e) holes & scratches: (d) holes, (e) scratches; (f) rodent marks; (g) snake marks; (h) crab marks; (I) lizard marks; (j-l) unknown predation marks. (DOCX)

S2 Fig Mean dorsal and ventral reflectance spectra including standard deviation of *Oophaga pumilio* specimens from six study populations. Mean reflectance curves average spectra of 38 to 52 individuals (Sarapiquí: 40; Hitoy: 52; Río Gloria: 38; Tierra Oscura: 47; Colón: 40; Isla Solarte: 38). Reflectance curves show reflection of the frogs relative to white standard. Reflectances over 100% emerge due to the brilliant surface of the frogs´ skin, while the used white standard has a dull surface. In order to verify accuracy of reflectance curves, all spectra were visually controlled for oversaturation.
(DOCX)

S3 Fig Conspicuousness of clay model frogs on local substrates for four different observers. We calculated conspicuousness measurements (color and brightness contrast) of blue, red, green and yellow clay model frogs on all measured substrates of the respective population for four different observers (avian, snake, crab, *O*. *pumilio*). Circles of different colors represent means of calculated conspicuousness measurements of each clay color in each population for the respective observer.
(DOCX)

S1 Table Differences in color contrast between populations for four different observers. (DOCX)

S2 Table Differences in brightness contrast between populations for four different observers. (DOCX)

S3 Table Number of non-attacked and attacked clay model frogs of different coloration in six study populations. (DOCX)

S4 Table (a–g) Pairwise differences in attack rates among frog populations, clay frogs of different colors and clay frogs of local and non-local coloration. (DOCX)

S5 Table Correlation between risk of an attack on clay model frogs and the conspicuousness of local specimens of strawberry poison frogs. (DOCX)
