# Correction: An Analysis of Predator Selection to Affect Aposematic Coloration in a Poison Frog Species

**DOI:** 10.1371/journal.pone.0134628

**Published:** 2015-07-30

**Authors:** The PLOS ONE Staff

The email address for the first author, Corinna E. Dreher, is incorrect. The correct email address is: corinnadreher@gmail.com. The publisher apologizes for this error.

Fig 4 is incorrect. The authors have provided a corrected version here.

**Fig 4:**
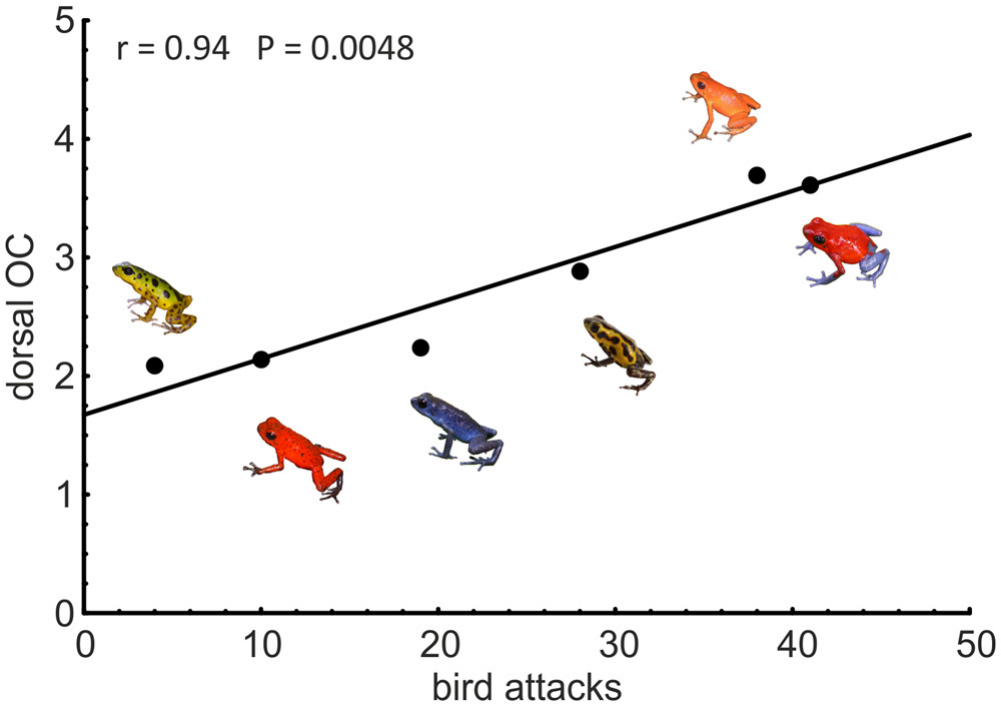
Correlation between dorsal overall conspicuousness of local frogs for avian eyes and avian predation on clay frogs across frog populations. The correlation is highly significant (r = Spearman rank correlation coefficient).
