# Supplementary figures and images for: An Analysis of Predator Selection to Affect Aposematic Coloration in a Poison Frog Species
Source: PLoS One. 2015 Jun 25;10(6):e0130571. doi: 10.1371/journal.pone.0130571 (PMC4481408; doi:10.1371/journal.pone.0130571)

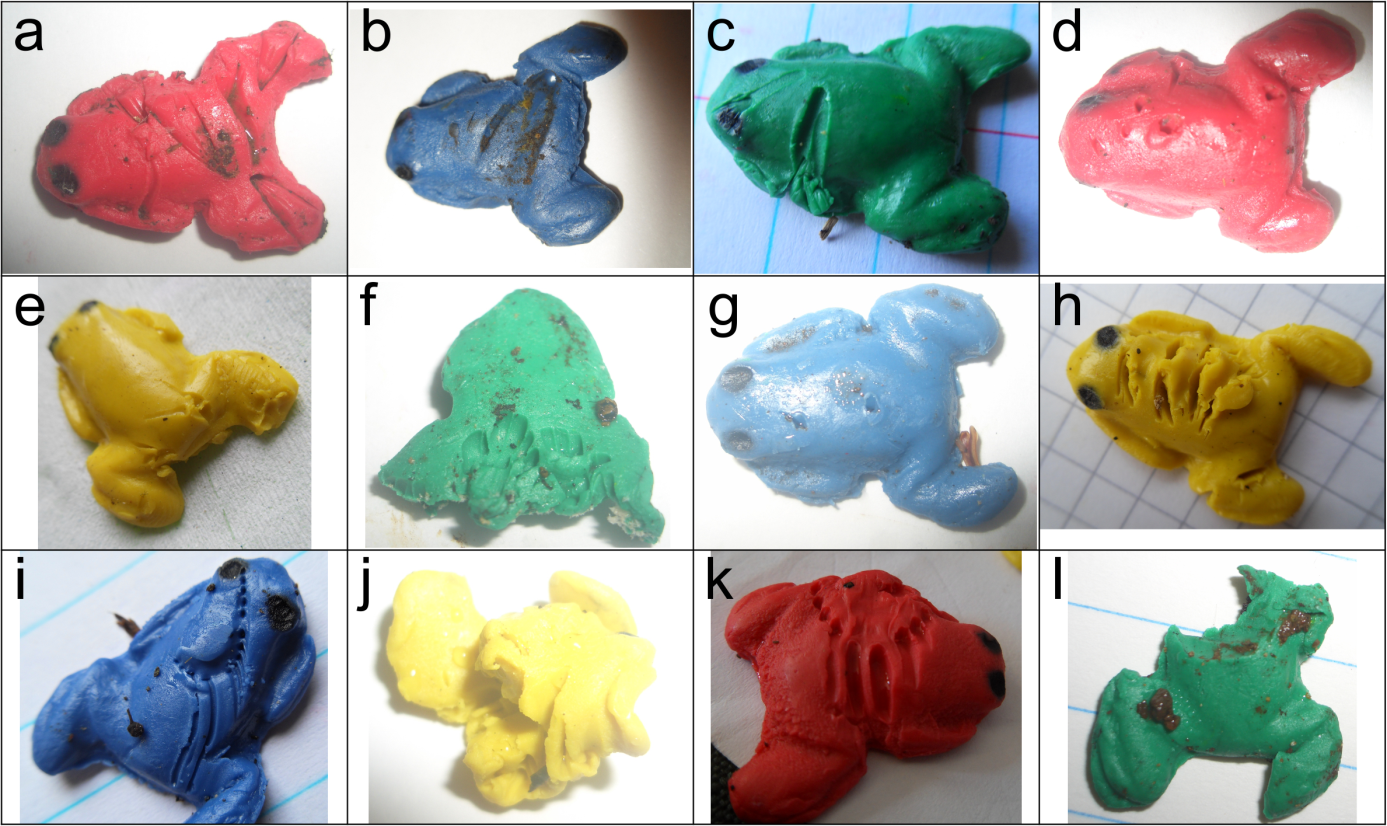

Supplement: S1 Fig — (DOCX) [file pone.0130571.s001.docx]

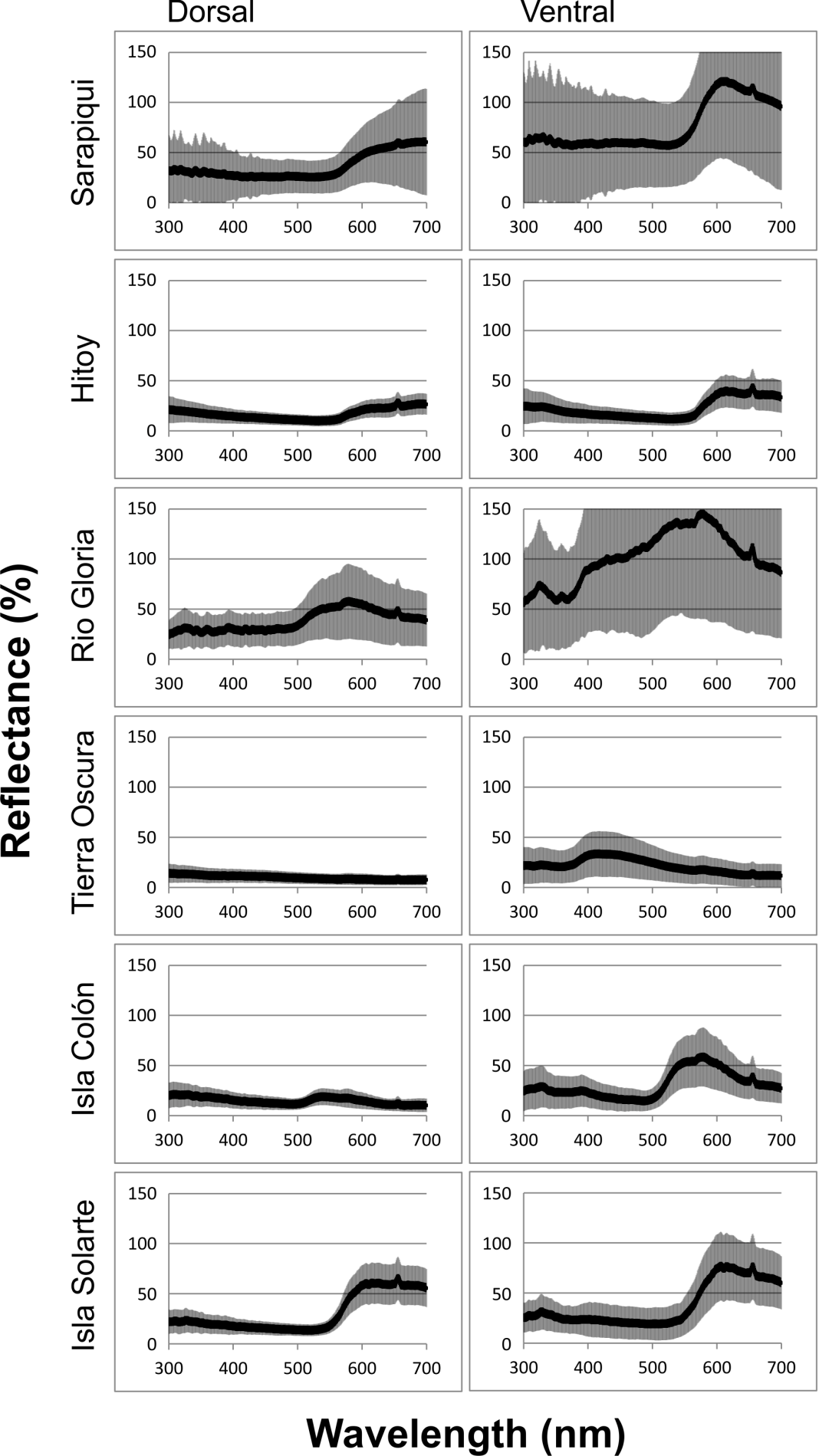

Supplement: S2 Fig — Mean reflectance curves average spectra of 38 to 52 individuals (Sarapiquí: 40; Hitoy: 52; Río Gloria: 38; Tierra Oscura: 47; Colón: 40; Isla Solarte: 38). Reflectance curves show reflection of the frogs relative to white standard. Reflectances over 100% emerge due to the brilliant surface of the frogs´ skin, while the used white standard has a dull surface. In order to verify accuracy of reflectance curves, all spectra were visually controlled for oversaturation. (DOCX) [file pone.0130571.s002.docx]

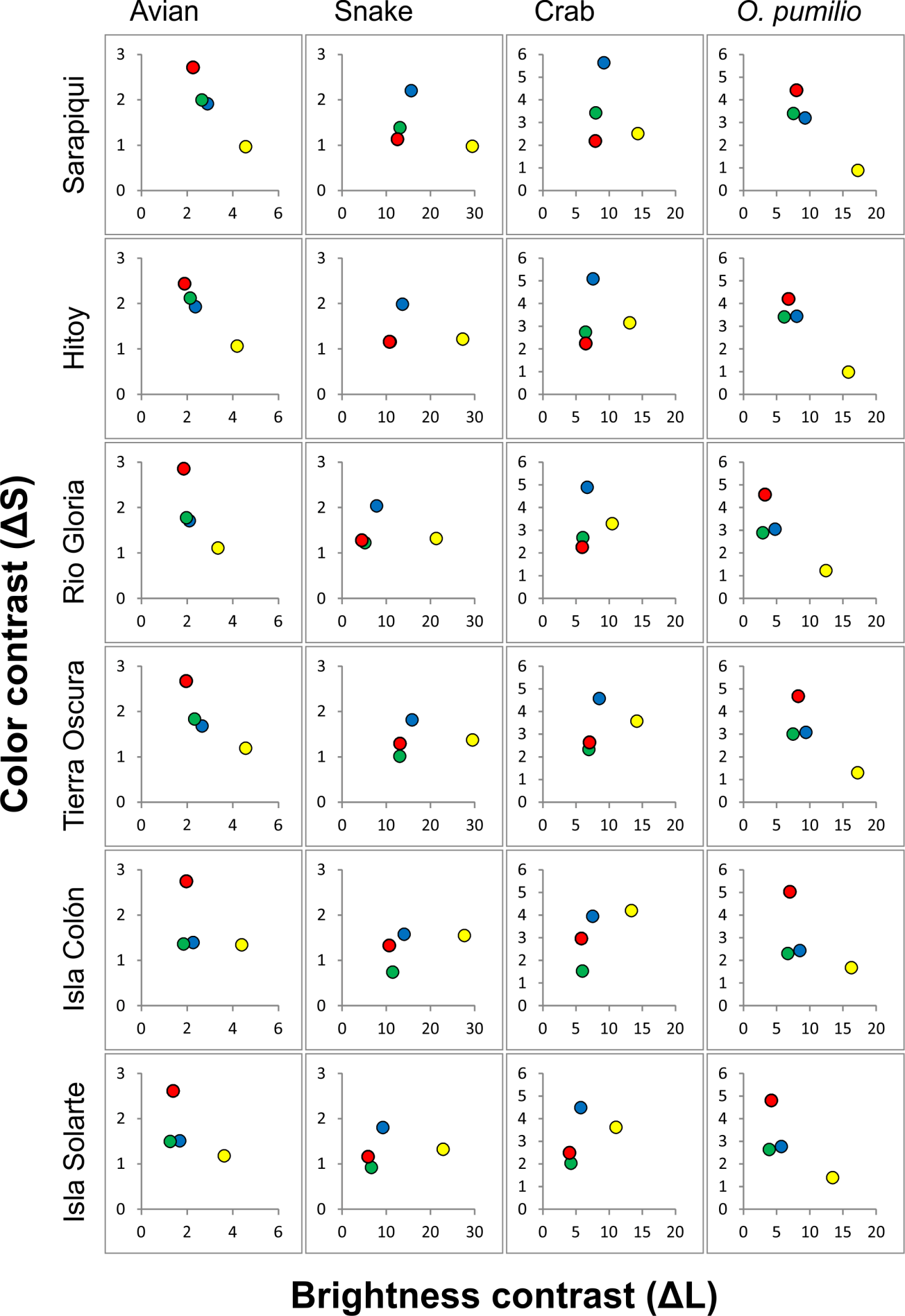

Supplement: S3 Fig — We calculated conspicuousness measurements (color and brightness contrast) of blue, red, green and yellow clay model frogs on all measured substrates of the respective population for four different observers (avian, snake, crab, O. pumilio). Circles of different colors represent means of calculated conspicuousness measurements of each clay color in each population for the respective observer. (DOCX) [file pone.0130571.s003.docx]
