# Supplementary material for: An Analysis of Predator Selection to Affect Aposematic Coloration in a Poison Frog Species
Source: PLoS One. 2015 Jun 25;10(6):e0130571. doi: 10.1371/journal.pone.0130571 (PMC4481408; doi:10.1371/journal.pone.0130571)
Supplement: S1 Table — (DOCX) [file pone.0130571.s004.docx]

**Table S1. Differences in color contrast between populations for four different observers.** Differences in color contrast between populations were localized using Tukey post-hoc test. P-values below 0.05 indicate that color contrasts between the respective two populations differ for the indicated observer.

DORSAL VENTRAL

|  | Sarapiqui | Hitoy C | Río Gloria | T.Oscura | I.Colón | I.Solarte |  | Sarapiqui | Hitoy C | Río Gloria | T.Oscura | I.Colón | I.Solarte |
| --- | --- | --- | --- | --- | --- | --- | --- | --- | --- | --- | --- | --- | --- |
|  | **Avian vision** | |  |  |  |  |  |  |  |  |  |  |  |
| Sarapiqui |  | 0.942 | 0.113 | 1.000 | 0.153 | **0.000** |  |  | 0.648 | 0.084 | 0.101 | 0.862 | **0.000** |
| Hitoy C | 0.942 |  | 0.473 | 0.930 | 0.570 | **0.000** |  | 0.648 |  | **0.000** | 0.852 | 1.000 | **0.000** |
| Río Gloria | 0.113 | 0.473 |  | 0.088 | 1.000 | **0.000** |  | 0.084 | **0.000** |  | **0.000** | **0.002** | **0.000** |
| T. Oscura | 1.000 | 0.930 | 0.088 |  | 0.122 | **0.000** |  | 0.101 | 0.852 | **0.000** |  | 0.731 | **0.000** |
| I.Colón | 0.153 | 0.570 | 1.000 | 0.122 |  | **0.000** |  | 0.862 | 1.000 | **0.002** | 0.731 |  | **0.000** |
| I.Solarte | **0.000** | **0.000** | **0.000** | **0.000** | **0.000** |  |  | **0.000** | **0.000** | **0.000** | **0.000** | **0.000** |  |
|  | **Snake vision** | | |  |  |  |  |  |  |  |  |  |  |
| Sarapiqui |  | 0.988 | 0.590 | **0.001** | 1.000 | **0.000** |  |  | 0.968 | 0.991 | **0.000** | **0.000** | **0.003** |
| Hitoy C | 0.988 |  | 0.888 | **0.003** | 1.000 | **0.000** |  | 0.968 |  | 0.712 | **0.000** | **0.000** | **0.000** |
| Río Gloria | 0.590 | 0.888 |  | 0.182 | 0.777 | **0.021** |  | 0.991 | 0.712 |  | **0.000** | **0.000** | **0.024** |
| T. Oscura | **0.001** | **0.003** | 0.182 |  | **0.002** | 0.929 |  | **0.000** | **0.000** | **0.000** |  | **0.036** | 0.740 |
| I. Colón | 1.000 | 1.000 | 0.777 | **0.002** |  | **0.000** |  | **0.000** | **0.000** | **0.000** | **0.036** |  | **0.000** |
| I. Solarte | **0.000** | **0.000** | **0.021** | 0.929 | **0.000** |  |  | **0.003** | **0.000** | **0.024** | 0.740 | **0.000** |  |
|  | **Crab vision** | |  |  |  |  |  |  |  |  |  |  |  |
| Sarapiqui |  | 1.000 | 0.718 | **0.000** | 1.000 | **0.000** |  |  | 0.838 | 1.000 | **0.000** | **0.000** | **0.000** |
| Hitoy C | 1.000 |  | 0.721 | **0.000** | 1.000 | **0.000** |  | 0.838 |  | 0.905 | **0.000** | **0.000** | **0.000** |
| Río Gloria | 0.718 | 0.721 |  | 0.088 | 0.612 | **0.000** |  | 1.000 | 0.905 |  | **0.000** | **0.000** | **0.000** |
| T. Oscura | **0.000** | **0.000** | 0.088 |  | **0.000** | 0.378 |  | **0.000** | **0.000** | **0.000** |  | 0.222 | 0.998 |
| I. Colón | 1.000 | 1.000 | 0.612 | **0.000** |  | **0.000** |  | **0.000** | **0.000** | **0.000** | 0.222 |  | 0.117 |
| I. Solarte | **0.000** | **0.000** | **0.000** | 0.378 | **0.000** |  |  | **0.000** | **0.000** | **0.000** | 0.998 | 0.117 |  |
|  | **Frog vision** | |  |  |  |  |  |  |  |  |  |  |  |
| Sarapiqui |  | 0.979 | 1.000 | 0.083 | 0.081 | **0.000** |  |  | 0.945 | **0.040** | **0.003** | 0.125 | **0.000** |
| Hitoy C | 0.979 |  | 0.999 | **0.004** | 0.279 | **0.000** |  | 0.945 |  | **0.001** | 0.029 | 0.499 | **0.000** |
| Río Gloria | 1.000 | 0.999 |  | **0.039** | 0.179 | **0.000** |  | **0.040** | **0.001** |  | **0.000** | **0.000** | **0.000** |
| T. Oscura | 0.083 | **0.004** | **0.039** |  | **0.000** | **0.000** |  | **0.003** | 0.029 | **0.000** |  | 0.867 | **0.000** |
| I. Colón | 0.081 | 0.279 | 0.179 | **0.000** |  | **0.000** |  | 0.125 | 0.499 | **0.000** | 0.867 |  | **0.000** |
| I. Solarte | **0.000** | **0.000** | **0.000** | **0.000** | **0.000** |  |  | **0.000** | **0.000** | **0.000** | **0.000** | **0.000** |  |
