# Supplementary material for: An Analysis of Predator Selection to Affect Aposematic Coloration in a Poison Frog Species
Source: PLoS One. 2015 Jun 25;10(6):e0130571. doi: 10.1371/journal.pone.0130571 (PMC4481408; doi:10.1371/journal.pone.0130571)
Supplement: S2 Table — (DOCX) [file pone.0130571.s005.docx]

**Table S2. Differences in brightness contrast between populations for four different observers.**

Differences in brightness contrast between populations were localized using Tukey post-hoc test. P-values below 0.05 indicate that brightness contrasts between the respective two populations differ for the indicated observer.

DORSAL VENTRAL

|  | Sarapiqui | Hitoy C | Río Gloria | T.Oscura | I.Colón | I.Solarte |  | Sarapiqui | Hitoy C | Río Gloria | T.Oscura | I.Colón | I.Solarte |
| --- | --- | --- | --- | --- | --- | --- | --- | --- | --- | --- | --- | --- | --- |
|  | **Avian vision** | |  |  |  |  |  |  |  |  |  |  |  |
| Sarapiqui |  | **0.000** | 0.291 | **0.000** | **0.000** | 0.053 |  |  | **0.000** | 0.438 | **0.000** | **0.000** | **0.000** |
| Hitoy C | **0.000** |  | **0.049** | 1.000 | 1.000 | 0.299 |  | **0.000** |  | **0.000** | 0.817 | 0.096 | 0.965 |
| Río Gloria | 0.291 | **0.049** |  | 0.077 | 0.124 | 0.979 |  | 0.438 | **0.000** |  | **0.000** | **0.016** | **0.000** |
| T. Oscura | **0.000** | 1.000 | 0.077 |  | 1.000 | 0.386 |  | **0.000** | 0.817 | **0.000** |  | **0.003** | 0.378 |
| I. Colón | **0.000** | 1.000 | 0.124 | 1.000 |  | 0.488 |  | **0.000** | 0.096 | **0.016** | **0.003** |  | 0.563 |
| I. Solarte | 0.053 | 0.299 | 0.979 | 0.386 | 0.488 |  |  | **0.000** | 0.965 | **0.000** | 0.378 | 0.563 |  |
|  |  |  |  |  |  |  |  |  |  |  |  |  |  |
|  | **Snake vision** | | |  |  |  |  |  |  |  |  |  |  |
| Sarapiqui |  | **0.000** | 0.496 | **0.000** | **0.000** | **0.016** |  |  | **0.000** | 0.726 | **0.000** | **0.000** | **0.000** |
| Hitoy C | **0.000** |  | **0.031** | 1.000 | 1.000 | 0.694 |  | **0.000** |  | **0.000** | 0.960 | **0.017** | 0.994 |
| Río Gloria | 0.496 | **0.031** |  | **0.047** | 0.082 | 0.697 |  | 0.726 | **0.000** |  | **0.000** | **0.022** | **0.000** |
| T. Oscura | **0.000** | 1.000 | **0.047** |  | 1.000 | 0.760 |  | **0.000** | 0.960 | **0.000** |  | **0.001** | 0.776 |
| I. Colón | **0.000** | 1.000 | 0.082 | 1.000 |  | 0.838 |  | **0.000** | **0.017** | **0.022** | **0.001** |  | 0.140 |
| I. Solarte | **0.016** | 0.694 | 0.697 | 0.760 | 0.838 |  |  | **0.000** | 0.994 | **0.000** | 0.776 | 0.140 |  |
|  |  |  |  |  |  |  |  |  |  |  |  |  |  |
|  | **Crab vision** | |  |  |  |  |  |  |  |  |  |  |  |
| Sarapiqui |  | **0.000** | 0.292 | **0.000** | **0.000** | **0.023** |  |  | **0.000** | 0.538 | **0.000** | **0.000** | **0.000** |
| Hitoy C | **0.000** |  | **0.045** | 1.000 | 1.000 | 0.466 |  | **0.000** |  | **0.000** | 0.929 | 0.119 | 0.989 |
| Río Gloria | 0.292 | **0.045** |  | 0.052 | 0.133 | 0.915 |  | 0.538 | **0.000** |  | **0.000** | **0.006** | **0.000** |
| T. Oscura | **0.000** | 1.000 | 0.052 |  | 1.000 | 0.483 |  | **0.000** | 0.929 | **0.000** |  | **0.010** | 0.662 |
| I. Colón | **0.000** | 1.000 | 0.133 | 1.000 |  | 0.698 |  | **0.000** | 0.119 | **0.006** | **0.010** |  | 0.500 |
| I. Solarte | **0.023** | 0.466 | 0.915 | 0.483 | 0.698 |  |  | **0.000** | 0.989 | **0.000** | 0.662 | 0.500 |  |
|  |  |  |  |  |  |  |  |  |  |  |  |  |  |
|  | **Frog vision** | |  |  |  |  |  |  |  |  |  |  |  |
| Sarapiqui |  | **0.000** | 0.293 | **0.000** | **0.000** | **0.039** |  |  | **0.000** | 0.476 | **0.000** | **0.000** | **0.000** |
| Hitoy C | **0.000** |  | **0.047** | 1.000 | 1.000 | 0.358 |  | **0.000** |  | **0.000** | 0.860 | 0.084 | 0.976 |
| Río Gloria | 0.293 | **0.047** |  | 0.075 | 0.124 | 0.961 |  | 0.476 | **0.000** |  | **0.000** | 0.015 | **0.000** |
| T. Oscura | **0.000** | 1.000 | 0.075 |  | 1.000 | 0.450 |  | **0.000** | 0.860 | **0.000** |  | **0.003** | 0.470 |
| I. Colón | **0.000** | 1.000 | 0.124 | 1.000 |  | 0.562 |  | **0.000** | 0.084 | 0.015 | **0.003** |  | 0.491 |
| I. Solarte | **0.039** | 0.358 | 0.961 | 0.450 | 0.562 |  |  | **0.000** | 0.976 | **0.000** | 0.470 | 0.491 |  |
