# Supplementary material for: An Analysis of Predator Selection to Affect Aposematic Coloration in a Poison Frog Species
Source: PLoS One. 2015 Jun 25;10(6):e0130571. doi: 10.1371/journal.pone.0130571 (PMC4481408; doi:10.1371/journal.pone.0130571)
Supplement: S3 Table — (DOCX) [file pone.0130571.s006.docx]

**Table S3. Number of non-attacked and attacked clay model frogs of different coloration in six study populations.** Attacked clay model frogs were assigned to one of the categories ‘bird marks’, ‘holes and scratches’, ‘rodent’, ‘snake’, ‘crab’, ‘lizard’, ‘missing’ or ‘unknown’. For statistical analyses we summarized the attacks of the categories ‘bird marks’ and ‘holes and scratches’ to the category ‘potential bird marks’ and all attacks of further categories to the category ‘non-bird predation’.

| **Population** | clay model color | origin | non-attacked | attacked | bird | holes & scratches | potential bird marks | rodent | snake | crab | lizard | missing | unknown | non-bird predation |
| --- | --- | --- | --- | --- | --- | --- | --- | --- | --- | --- | --- | --- | --- | --- |
|  | red | local | 382 | 18 | 11 | 7 | 18 | 0 | 0 | 0 | 0 | 0 | 0 | 0 |
| **Sarapiqui** | blue | non-local | 367 | 33 | 19 | 13 | 32 | 1 | 0 | 0 | 0 | 0 | 0 | 1 |
| **(red)** | yellow | non-local | 385 | 15 | 5 | 6 | 11 | 1 | 0 | 0 | 0 | 0 | 3 | 4 |
|  | green | non-local | 385 | 15 | 3 | 10 | 13 | 1 | 0 | 0 | 0 | 0 | 1 | 2 |
|  | red | local | 381 | 19 | 2 | 14 | 16 | 2 | 0 | 0 | 0 | 0 | 1 | 3 |
| **Hitoy Cerere** | blue | non-local | 375 | 25 | 5 | 17 | 22 | 0 | 0 | 0 | 2 | 0 | 1 | 3 |
| **(red)** | yellow | non-local | 382 | 18 | 0 | 13 | 13 | 1 | 0 | 1 | 0 | 1 | 2 | 5 |
|  | green | non-local | 383 | 17 | 3 | 14 | 17 | 0 | 0 | 0 | 0 | 0 | 0 | 0 |
|  | red | non-local | 373 | 27 | 5 | 18 | 23 | 2 | 0 | 0 | 0 | 2 | 0 | 4 |
| **Río Gloria** | blue | non-local | 366 | 34 | 13 | 13 | 26 | 4 | 0 | 0 | 0 | 3 | 1 | 8 |
| **(yellow)** | yellow | local | 381 | 19 | 3 | 13 | 16 | 2 | 0 | 0 | 0 | 1 | 0 | 3 |
|  | green | non-local | 377 | 23 | 7 | 13 | 20 | 1 | 0 | 0 | 0 | 1 | 1 | 3 |
|  | red | non-local | 382 | 18 | 4 | 11 | 15 | 0 | 0 | 0 | 0 | 1 | 2 | 3 |
| **Tierra Oscura** | blue | local | 379 | 21 | 4 | 17 | 21 | 0 | 0 | 0 | 0 | 0 | 0 | 0 |
| **(blue)** | yellow | non-local | 382 | 18 | 6 | 10 | 16 | 0 | 0 | 0 | 0 | 0 | 2 | 2 |
|  | green | non-local | 380 | 20 | 5 | 15 | 20 | 0 | 0 | 0 | 0 | 0 | 0 | 0 |
|  | red | non-local | 371 | 29 | 0 | 21 | 21 | 0 | 2 | 2 | 0 | 0 | 4 | 8 |
| **Isla Colón** | blue | non-local | 368 | 32 | 1 | 25 | 26 | 2 | 2 | 0 | 0 | 1 | 1 | 6 |
| **(green)** | yellow | non-local | 371 | 29 | 0 | 26 | 26 | 1 | 1 | 0 | 0 | 0 | 1 | 3 |
|  | green | local | 367 | 33 | 3 | 27 | 30 | 0 | 0 | 0 | 0 | 1 | 2 | 3 |
|  | red | local | 360 | 40 | 14 | 23 | 37 | 2 | 0 | 0 | 0 | 1 | 0 | 3 |
| **Isla Solarte** | blue | non-local | 360 | 40 | 11 | 24 | 35 | 3 | 2 | 0 | 0 | 0 | 0 | 5 |
| **(red)** | yellow | non-local | 366 | 34 | 12 | 21 | 33 | 1 | 0 | 0 | 0 | 0 | 0 | 1 |
|  | green | non-local | 364 | 36 | 4 | 29 | 33 | 3 | 0 | 0 | 0 | 0 | 0 | 3 |
