# Supplementary material for: An Analysis of Predator Selection to Affect Aposematic Coloration in a Poison Frog Species
Source: PLoS One. 2015 Jun 25;10(6):e0130571. doi: 10.1371/journal.pone.0130571 (PMC4481408; doi:10.1371/journal.pone.0130571)
Supplement: S4 Table — (DOCX) [file pone.0130571.s007.docx]

**Table S4a. Differences in for overall attack rate between populations.** Results (P-values) of Generalized linear models (GLM). P-values < 0.05 indicate differences between populations.

|  | Sarapiqui | Hitoy Cerere | Río Gloria | Tierra Oscura | Isla Colón | Isla Solarte |
| --- | --- | --- | --- | --- | --- | --- |
| Sarapiqui |  |  |  |  |  |  |
| Hitoy Cerere | 1 |  |  |  |  |  |
| Río Gloria | 0.5524 | 0.4467 |  |  |  |  |
| Tierra Oscura | 0.9995 | 1.0000 | 0.3509 |  |  |  |
| Isla Colón | **0.0301** | **0.0185** | 0.7371 | **0.0113** |  |  |
| Isla Solarte | **<0.001** | **<0.001** | 0.261 | **<0.001** | 0.5239 |  |

**Table S4b. Differences in ‘overall attack rate’ between different colors of clay models and between clay models of local and non-local coloration for all populations (*O. pumilio*) and for each population.** P-values (Tukey-Test) below 0.05 indicate that attack rate differed between the two compared colors, or models of local and non-local origin (last column).

|  | blue-yellow | blue-green | blue-red | yellow-green | yellow-red | green-red | local-nonlocal |
| --- | --- | --- | --- | --- | --- | --- | --- |
| *O.pumilio* | **0.014** | 0.0906 | 0.2773 | 0.9029 | 0.6964 | 0.9728 | 0.921 |
| Sarapiqui | **0.044** | **0.0441** | 0.1392 | 1 | 0.951 | 0.9507 | 0.553877 |
| Hitoy Cerere | 0.6935 | 0.5875 | 0.7896 | 0.9982 | 0.9983 | 0.9863 | 0.8416 |
| Río Gloria | 0.151 | 0.4345 | 0.7877 | 0.921 | 0.6199 | 0.9368 | 0.11456 |
| Tierra Oscura | 0.9609 | 0.9985 | 0.9609 | 0.9873 | 1 | 0.9873 | 0.63707 |
| Isla Colón | 0.9784 | 0.9992 | 0.9784 | 0.9522 | 1 | 0.9522 | 0.62593 |
| Isla Solarte | 0.8845 | 0.9631 | 1 | 0.9945 | 0.8845 | 0.9631 | 0.62058 |

**Table S4c. Differences attack rate among populations in the category ‘bird marks’.** Results (P-values) of generalized linear models (GLM). P-values < 0.05 indicate differences between populations.

|  | Sarapiqui | Hitoy Cerere | Río Gloria | Tierra Oscura | Isla Colón | Isla Solarte |
| --- | --- | --- | --- | --- | --- | --- |
| Sarapiqui |  |  |  |  |  |  |
| Hitoy Cerere | **0.00173** |  |  |  |  |  |
| Río Gloria | 0.81145 | **0.04838** |  |  |  |  |
| Tierra Oscura | 0.1128 | 0.5576 | 0.74964 |  |  |  |
| Isla Colón | **<0.001** | 0.6135 | **0.00311** | **0.04948** |  |  |
| Isla Solarte | 0.99931 | **<0.001** | 0.60529 | 0.05042 | **<0.001** |  |

**Table S4d. Differences in attack rate in the category ‘bird marks’ on different colors of clay models and on clay models of local and non-local coloration for summarized results of all populations (*O. pumilio*) and for each population.** P-values (Tukey-Test) below 0.05 indicate that attack rate differed between the two compared colors, or models of local and non-local coloration (last column).

|  | blue-yellow | blue-green | blue-red | yellow-green | yellow-red | green-red | local-nonlocal |
| --- | --- | --- | --- | --- | --- | --- | --- |
| *O. pumilio* | **0.0135** | **0.0108** | 0.3162 | 0.9996 | 0.682 | 0.6644 | 0.819 |
| Sarapiqui | **0.0326** | **0.0128** | 0.443 | 0.8915 | 0.4391 | 0.1746 | 0.57017 |
| Hitoy Cerere | 1 | 0.8757 | 0.644 | 1 | 1 | 0.9641 | 0.7151 |
| Río Gloria | 0.0916 | 0.5315 | 0.2495 | 0.597 | 0.8933 | 0.9367 | 0.0917 |
| Tierra Oscura | 0.9215 | 0.9871 | 1 | 0.9903 | 0.9215 | 0.9871 | 0.69 |
| Isla Colón | 1 | 0.7306 | 1 | 1 | 1 | 1 | 0.05662 |
| Isla Solarte | 0.9966 | 0.2918 | 0.9279 | 0.2129 | 0.9781 | 0.1101 | 0.1741 |

**Table S4e. Differences attack rate among populations in the category ‘potential bird marks’.** Results (P-values) of generalized linear models (GLM). P-values < 0.05 indicate differences between populations.

|  | Sarapiqui | Hitoy Cerere | Río Gloria | Tierra Oscura | Isla Colón | Isla Solarte |
| --- | --- | --- | --- | --- | --- | --- |
| Sarapiqui |  |  |  |  |  |  |
| Hitoy Cerere | 0.9956 |  |  |  |  |  |
| Río Gloria | 0.9452 | 0.7161 |  |  |  |  |
| Tierra Oscura | 1.0000 | 0.9995 | 0.8823 |  |  |  |
| Isla Colón | 0.2224 | 0.6898 | 0.7624 | 0.1482 |  |  |
| Isla Solarte | **<0.001** | **<0.001** | **0.0038** | **<0.001** | 0.1755 |  |

**Table S4f. Differences of attack rate in the category ‘potential bird marks’ on different colors of clay models and on clay models of local and non-local coloration for summarized results of all populations (*O. pumilio*) and for each population.** P-values (Tukey-Test) below 0.05 indicate that attack rate differed between the two compared colors, or models of local and non-local coloration (last column).

|  | blue-yellow | blue-green | blue-red | yellow-green | yellow-red | green-red | local-nonlocal |
| --- | --- | --- | --- | --- | --- | --- | --- |
| *O.pumilio* | **0.0089** | **0.0239** | 0.17826 | 0.97569 | 0.55161 | 0.79582 | 0.546 |
| Sarapiqui | 0.4133 | 0.8452 | 0.7526 | 0.8796 | 0.942 | 0.998 | 0.8907 |
| Hitoy Cerere | 0.3947 | 0.7996 | 0.971 | 0.9041 | 0.6619 | 0.9655 | 0.7748 |
| Río Gloria | 0.3947 | 0.7996 | 0.971 | 0.9041 | 0.6619 | 0.9655 | 0.1789 |
| Tierra Oscura | 0.8355 | 0.9985 | 0.7381 | 0.9042 | 0.9978 | 0.8244 | 0.4043 |
| Isla Colón | 1 | 0.9455 | 0.8764 | 0.9455 | 0.8764 | 0.565 | 0.3183 |
| Isla Solarte | 0.9943 | 0.9943 | 0.9947 | 1 | 0.959 | 0.959 | 0.6073 |

**Table S4g. Differences attack rate among populations in the category ‘non-bird marks’.** Results (P-values) of generalized linear models (GLM). P-values < 0.05 indicate differences between populations.

|  | Sarapiqui | Hitoy | Río Gloria | Tierra Oscura | Colón | Solarte |
| --- | --- | --- | --- | --- | --- | --- |
| Sarapiqui |  |  |  |  |  |  |
| Hitoy Cerere | 0.9343 |  |  |  |  |  |
| Río Gloria | 0.3072 | 0.8309 |  |  |  |  |
| Tierra Oscura | 0.9912 | 0.6689 | 0.1207 |  |  |  |
| Isla Colón | 0.2244 | 0.7280 | 1 | 0.0824 |  |  |
| Isla Solarte | 0.8614 | 0.9999 | 0.9147 | 0.547 | 0.8377 |  |

**Table S4h. Differences of attack rate in the category ‘non-bird predation’ on different colors of clay models and on clay models of local and non-local coloration for summarized results of all populations (*O. pumilio*) and for each population.** P-values (Tukey-Test) below 0.05 indicate that attack rate differed between the two compared colors, or models of local and non-local coloration (last column).

|  | blue-yellow | blue-green | blue-red | yellow-green | yellow-red | green-red | local-nonlocal |
| --- | --- | --- | --- | --- | --- | --- | --- |
| *O.pumilio* | 0.952 | 0.286 | 0.995 | 0.952 | 0.286 | 0.995 | 0.102 |
| Sarapiqui | 0.5453 | 0.9292 | 1 | 0.8247 | 1 | 1 | 0.9986 |
| Hitoy Cerere | 0.8762 | 1 | 1 | 1 | 0.8762 | 1 | 0.8614 |
| Río Gloria | 0.4579 | 0.458 | 0.6605 | 1 | 0.9812 | 0.9812 | 0.4166 |
| Tierra Oscura | 1 | 1 | 1 | 1 | 0.9625 | 1 | 0.9978 |
| Isla Colón | 0.7538 | 0.7538 | 0.949 | 1 | 0.457 | 0.4571 | 0.3068 |
| Isla Solarte | 0.4433 | 0.8927 | 0.8927 | 0.7695 | 0.7695 | 1 | 1 |
