# Supplementary material for: An Analysis of Predator Selection to Affect Aposematic Coloration in a Poison Frog Species
Source: PLoS One. 2015 Jun 25;10(6):e0130571. doi: 10.1371/journal.pone.0130571 (PMC4481408; doi:10.1371/journal.pone.0130571)
Supplement: S5 Table — (DOCX) [file pone.0130571.s008.docx]

**Table S5. Correlation between risk of an attack on clay model frogs and the conspicuousness of local specimens of strawberry poison frogs.** Correlational analyses were calculated to test for a relationship of ‘overall attack rate’, ‘bird marks’, ‘potential bird marks’ and ‘non-bird predation’ with the conspicuousness (color and brightness contrast) of local, living specimens of *O. pumilio*.

|  | avian ∆S | avian ∆L | crab ∆S | crab ∆L | snake ∆S | snake ∆L |
| --- | --- | --- | --- | --- | --- | --- |
| overall attack rate | .6288 | -.0143 | .4256 | -.1005 | .4764 | -.0457 |
|  | p=.181 | p=.979 | p=.400 | p=.850 | p=.340 | p=.932 |
| bird marks | .6373 | .7919 |  |  |  |  |
|  | p=.173 | p=.060 |  |  |  |  |
| potential bird marks | .7539 | .0179 |  |  |  |  |
|  | p=.083 | p=.973 |  |  |  |  |
| non-bird predation |  |  | -.3050 | -.1271 | -.2931 | -.1194 |
|  |  |  | p=.557 | p=.810 | p=.573 | p=.822 |
